# Honouring Rinaldo Bellomo’s tour de force in sepsis research

**DOI:** 10.1016/j.ccrj.2025.100139

**Published:** 2025-10-14

**Authors:** S. Peake, A. Delaney, B. Venkatesh

**Affiliations:** a Department of Intensive Care Medicine, The Queen Elizabeth Hospital, Adelaide, South Australia, Australia; b Discipline of Acute Care Medicine, The University of Adelaide, Adelaide, South Australia, Australia; c Australian and New Zealand Intensive Care Research Centre, School of Public Health and Preventive Medicine, Monash University, Melbourne, Victoria, Australia; d The George Institute for Global Health, Sydney, New South Wales, Australia; e Intensive Care, Royal North Shore Hospital, St Leonards, New South Wales, Australia; f Gold Coast University Hospital, Southport, Queensland, Australia

## Abstract

This paper pays tribute to Rinaldo Bellomo’s unparallelled global legacy in sepsis research. As a clinician-scientist, Rinaldo’s unending quest was to improve the lives of our patients with sepsis and septic shock. The breadth of his work encompassed all facets of research methodology from basic science through to large-scale multicentre randomised trials. Seminal papers like the early goal-directed therapy and steroids in septic shock trials have been instrumental in informing international evidence-based sepsis guidelines and changing clinical practice. Above all, he was a pioneering, yet self-effacing, leader. He brought everyone who worked with him along on his journey. His infectious enthusiasm for the next new idea never failed to inspire our community, ensuring that his legacy in sepsis research will continue for many years to come.

*“The teacher who is indeed wise does not bid you to enter the house of his wisdom but rather leads you to the threshold of your mind”. - Khalil Gibran*

What a journey! Over the past three decades Rinaldo’s sepsis research has encompassed every facet of research methodology, from animal models to observational studies to multicentre randomised trials, and finally, to international evidence-based guidelines that have informed global practice on how best to manage our patients with septic shock.^1^

In all, Rinaldo has authored over 400 publications on sepsis, the first of which was a case report published in 1992 describing dog-bite-induced *Capnocytophaga canimorsus* septicaemia.^2^ In the 1990s, much of his sepsis research involved preclinical models of Gram-negative shock, initially partnering with John Kellum at the University of Pittsburgh Medical Centre (USA) on a canine endotoxaemic model which provided important insights into the mechanisms of sepsis-related organ hypoperfusion,^3^ acid–base disturbance,^4^^,^^5^^,^^6^ and the effects of noradrenaline on renal blood flow.^7^ After his return to Australia, Rinaldo teamed with Clive May at the Preclinical Care Unit, Florey Institute of Neuroscience and Mental Health, University of Melbourne, to successfully establish a live *Escherichia coli* model of hyperdynamic ovine septic shock. He continued to study this clinically relevant and informative animal model over the next two decades, particularly focusing on altered regional blood flow distribution and the pathogenesis and management of sepsis-induced acute renal failure while also mentoring and collaborating with multiple preclinical and early-career researchers.^8^^,^^9^^,^^10^^,^^11^

Alongside his basic science research, Rinaldo published two seminal papers describing the burden of sepsis in Australian and New Zealand intensive care units (ICUs).^12^^,^^13^ His 2014 retrospective, an epidemiologic study published in *JAMA* included 101,304 patients with severe sepsis from 171 Australian and New Zealand ICUs utilising the binational Australian and New Zealand Intensive Care Society Adult Patient Database (APD). While hospital mortality was found to have decreased from 35% in 2000 to 18% in 2012, this research highlighted the need to conduct large-scale multicentre randomised trials evaluating new therapies to improve the outcome of patients with septic shock not only across our two countries but also worldwide. The importance of conducting our own investigator-initiated sepsis trials, rather than relying on evidence generated overseas, was further emphasised by Rinaldo’s analysis of Adult Patient Database data demonstrating that severity-adjusted mortality rates following admission to the ICU in Australia and New Zealand, including for sepsis, are better than usual care in other ICUs worldwide.^14^

Rinaldo conducted multiple other sepsis-related observational studies throughout his research career, many of which became the precursors for large, multicentre randomised trials. Some of these observational studies included renin and angiotensin II levels in septic shock,^15^ clinical characteristics,^16^ perfusion pressure,^17^ and neutrophil gelatinase–associated lipocalin levels in septic acute kidney injury. Mortality prediction using circulating biomarkers such as ferritin,^18^ cortisol, aldosterone, and ascorbic acid^19^ was also investigated, as was the timing of adjunctive vasopressin administration.[17], [20]

Another observational, inception cohort study examining early haemodynamic resuscitation practices in Australia and New Zealand, the Australasian Resuscitation In Sepsis Evaluation (ARISE) survey, was pivotal for the design, NH&MRC funding, and conduct of the ARISE multinational, randomised trial of early goal directed therapy (EGDT) in patients presenting to the emergency department with septic shock.^21^ In 2006, in the nascent years of the Australian and New Zealand Intensive Care Society Clinical Trials Group (ANZICS CTG), Rinaldo proposed the idea of a large, multicentre, randomised trial that would provide high-quality evidence for a novel haemodynamic strategy that had been rapidly incorporated into the international surviving sepsis campaign guidelines on the strength of a small, single-centre trial of EGDT in early septic shock^22^; the impetus for this question being that hospital mortality with usual care in Australia and New Zealand was lower than that reported in both the usual care and intervention arms of the then practice-changing EGDT trial conducted in the United States.^14^

In true Rinaldo fashion, he asked the audience at the 8th Annual ANZICS CTG Scientific Meeting who wanted to be a part of this journey with him? So began two decades of research into haemodynamic resuscitation in early septic shock. The CTG-endorsed ARISE trial,^23^ published in the *New England Journal of Medicine* in 2014, became one of a trilogy of collaborative and prospectively harmonised EGDT studies with Rinaldo’s colleagues at the University of Pittsburgh (ProCESS)^24^ and the UK Intensive Care and National Audit Research Centre (ProMISe).^25^ These collaborations culminated a few years later in an individual patient data meta-analysis, also published in the *New England Journal of Medicine*. In response, the surviving sepsis campaign guidelines changed to the no-longer-recommend EGDT as part of the initial resuscitation package for patients with septic shock.^26^

Rinaldo’s ability to nurture and maintain international and local collaborations was characterised by his multiple publications dating back over 20 years with Claudio Ronco at St Bortolo Hospital, Vincenza.^27^^,^^28^^,^^29^ Their work on sepsis-associated acute kidney injury, cytokines, and renal replacement therapy has provided important insights into the pathophysiology and management of septic shock and organ dysfunction. A successful partnership with Giovanni Landoni at San Raffaele Scientific Institute, Milan, also saw the publication in *JAMA* of the investigator-initiated MERCY study, a double-blind, randomised trial examining intermittent versus continuous meropenem infusion in critically ill patients with sepsis in 31 ICUs across four countries.^30^^,^^31^^,^^32^ The MERCY trial did not find a clinical benefit for continuous infusion (composite outcome of mortality and emergence of drug-resistant bacteria at day 28).

At a local level, Rinaldo teamed with other CTG investigators and the Burns, Trauma and Critical Care Research Centre, The University of Queensland, to study a similar question of how best to optimise antimicrobial therapy in severe sepsis. The results of the Beta-Lactam Infusion Group (BLING) II multicentre, randomised trial of continuous versus intermittent β-lactam administration were essential for the design, NH&MRC funding, and conduct of the definitive, randomised, CTG-endorsed BLING-III trial in 7202 critically ill patients with sepsis.^33^ The subsequent systematic review and meta-analysis found that the use of prolonged β-lactam infusions was associated with reduced 90-day mortality and increased probability of clinical cure, with a number needed to treat of 26 patients.^34^ Ongoing collaborations with Jason Roberts et al. at The University of Queensland have also provided a greater clinical understanding of the effects of renal replacement therapy on antimicrobial pharmacokinetics and pharmacodynamics, including the multinational Sampling Antibiotics in Renal Replacement Therapy (SMARRT) pharmacokinetic study which found that renal replacement therapy techniques and antibiotic dosing regimens result in highly variable antibiotic concentrations that often fail to achieve therapeutic drug targets in critically ill patients.^35^

Rinaldo also played a major role in the CTG becoming a world leader in corticosteroid research in sepsis through his seminal role in the CTG-endorsed ADRENAL trial.^36^ ADRENAL is one of the few trials where there has been significant knowledge gain about the role of corticosteroids ranging from clinical phenotype data to heterogeneity of treatment effect,^37^ to serum endocrine biomarkers,^38^ and finally, down to the level of the genome^39^ and the transcriptome. Rinaldo was pivotal to ADRENAL’s success at every stage—a chief investigator on the successful NHMRC grant, strong engagement in the management committee, data analysis and manuscript preparation, substudies, and partnership with Endpoint Health for transcriptomic evaluation. He was also instrumental in laying the foundations for a phase 3 trial of fludrocortisone.^40^

Rinaldo’s decades of ground-breaking, scientifically rigorous sepsis research continues today through the many ongoing clinical trials he has played a pivotal role in designing and conducting over the past two decades. The first interventional sepsis trial led by the CTG was made possible by Rinaldo.^41^ It was his idea to explore the role of statins in sepsis. This landmark phase 2 study was the launching springboard for the many sepsis trials to come from the CTG. Randomised trials examining EGDT (ARISE), vitamin C (VITAMINS), and angiotensin-II (ATHOS-3) administration have stimulated new research questions, new hypotheses, and new interventions to be tested. The Australasian Resuscitation In Sepsis Evaluation: FLUid or vasopressors In emergency Department Sepsis trial (ARISE FLUIDS), a 1000-patient, multinational, randomised trial comparing a restrictive fluid and early vasopressor strategy versus usual care in patients presenting to the emergency department with septic shock is due to complete recruitment later this year (Clinical Trials.gov NCT04569942). Like the ARISE EGDT trial, the results of the ARISE FLUIDS trial will inform global practice on haemodynamic resuscitation in early septic shock both within the emergency department and the ICU.

Intrigued by the concept of “metabolic resuscitation”, Rinaldo helped deliver the VITAMINS randomised, clinical trial published in *JAMA* in 2020.^42^ The VITAMINS trial reported no benefit of high-dose ascorbic acid in vasopressor-dependent septic shock. However, understanding a critical nuance in the trial intervention, Rinaldo realised that the dose and/or formulation may have been inadequate,^43^ thereby stimulating a novel program of preclinical research in a large animal model^44^ that has translated into clinical trials exploring the potential of megadoses of the base salt of vitamin C, sodium ascorbate, in septic shock.^45^

On the background of the multicentre randomised ATHOS-3 trial of angiotensin II for the treatment of vasodilatory shock published in the *New England Journal of Medicine* in 2017, Rinaldo has continued his research into angiotensin-II as an effective, potentially first-line vasoconstrictor. The ATHOS-3 trial found that continuous infusion of angiotensin-II could effectively augment mean arterial pressure in patients with catecholamine refractory shock. The ongoing phase 2b randomised trial led by Rinaldo examines the role of angiotensin II as a first-line vasoconstrictor versus noradrenaline in critically ill patients with vasodilatory shock (ACTRN1261001043820). Rinaldo’s quote that “By using Angiotensin II from the beginning, we aim to improve patient outcomes and reduce the risks associated with current treatments” exemplifies his unrelenting and ever-questioning approach to conducting high-quality, transformative research addressing key gaps in our knowledge of the pathophysiology, management, and outcomes of critically ill patients with septic shock.^46^ Finally, Rinaldo’s role as a mentor, including acting as an associate investigator for the National Critical Care Research Platform Medical Research Future Fund (MRFF) grant for early- and mid-career researchers to build capacity and infrastructure aimed at improving experiences and outcomes for ICU patients with sepsis also ensures that the next generation of researchers is equipped to continue his sepsis journey.

On a personal level, Rinaldo will be remembered in our research community as someone who was warm, welcoming, and inclusive. He was always open to new ideas, and his vision for always seeming to know the right questions to ask was unparallelled. He approached each new question with infectious enthusiasm, boundless energy, and a passion for making a difference. Without Rinaldo, the research careers of many within our community would not be where they are today. Thank you, Rinaldo.

## Funding

This research did not receive any specific grant from funding agencies in the public, commercial, or not-for-profit sectors.

## Credit authorship contribution statement

SP Conceptualisation, writing, original first draft, editing and final revision. AD Conceptualisation, writing, editing and final revision. BV Conceptualisation, writing, editing and final revision.

## Conflict of interest

The authors declare that they have no known competing financial interests or personal relationships that could have appeared to influence the work reported in this paper.
